# First-line salvage treatment options for germ cell tumor patients failing stage-adapted primary treatment: A comprehensive review compiled by the German Testicular Cancer Study Group

**DOI:** 10.1007/s00345-022-03959-8

**Published:** 2022-02-28

**Authors:** David Pfister, Karin Oechsle, Stefanie Schmidt, Jonas Busch, Carsten Bokemeyer, Axel Heidenreich, Julia Heinzelbecker, Christian Ruf, Christian Winter, Friedemann Zengerling, Sabine Kliesch, Peter Albers, Christoph Oing

**Affiliations:** 1 Department of Urology, University Hospital Cologne, Cologne, Germany; 2 Department of Oncology, Hematology and Bone Marrow Transplantation, University Medical Center Eppendorf, Hamburg, Germany; 3 UroEvidence@Deutsche Gesellschaft für Urologie, Berlin, Germany; 4 Department of Urology, Charité University Hospital, Campus Benjamin Franklin, Berlin, Germany; 5 Department of Urology and Paediatric Urology, Saarland University Medical Centre and Saarland University Faculty of Medicine, Homburg, Saar Germany; 6 Department of Urology, Bundeswehrkrankenhaus (Armed Forces Hospital, Ulm), Ulm, Germany; 7 Urologie Neandertal (Regional Joint Practice), Erkrath, Germany; 8 Department of Urology, University Hospital Ulm, Ulm, Germany; 9 Centre of Reproductive Medicine and Andrology, Department of Clinical and Surgical Andrology, University Hospital Muenster, Muenster, Germany; 10 Department of Urology, Medical Faculty, University Hospital Duesseldorf, Heinrich Heine University, Duesseldorf, Germany; 11 Mildred Scheel Cancer Career Center HaTriCs4, University Cancer Center Hamburg, University Medical Center Eppendorf, Hamburg, Germany; 12 Department of Urology, Uro-Oncology and Robot Assisted Surgery, University Hospital of Cologne, Kerpener Str. 62, 50937 Cologne, Germany

**Keywords:** Germ cell tumor, Relapse, Salvage treatment, High-dose chemotherapy, Salvage surgery

## Abstract

**Purpose:**

In this review, we summarize and discuss contemporary treatment standards and possible selection criteria for decision making after failure of adjuvant or first-line cisplatin-based chemotherapy for primarily localized or metastatic germ cell tumors.

**Methods:**

This work is based on a systematic literature search conducted for the elaboration of the first German clinical practice guideline to identify prospective clinical trials and retrospective comparative studies published between Jan 2010 and Feb 2021. Study end points of interest were progression-free (PFS) and overall survival (OS), relapse rate (RR), and/or safety.

**Results:**

Relapses of clinical stage I (CS I) patients irrespective of prior adjuvant treatment after orchiectomy are treated stage adapted in accordance for primary metastatic patients. Surgical approaches for sole retroperitoneal relapses are investigated in ongoing clinical trials. The appropriate salvage chemotherapy for metastatic patients progressing or relapsing after first-line cisplatin-based chemotherapy is still a matter of controversy. Conventional cisplatin-based chemotherapy is the international guideline-endorsed standard of care, but based on retrospective data high-dose chemotherapy and subsequent autologous stem cell transplantation may offer a 10–15% survival benefit for all patients. Secondary complete surgical resection of all visible residual masses irrespective of size is paramount for treatment success.

**Conclusions:**

Patients relapsing after definite treatment of locoregional disease are to be treated by stage-adapted first-line standard therapy for metastatic disease. Patients with primary advanced/metastatic disease failing one line of cisplatin-based combination chemotherapy should be referred to GCT expert centers. Dose intensity is a matter of ongoing debate, but sequential high-dose chemotherapy seems to improve patients’ survival.

## Introduction

Cancer-specific survival of clinical stage I (CS I) germ cell tumor (GCT) patients is excellent irrespective of the application of adjuvant treatment following orchiectomy and active surveillance for all patients is considered safe while omitting possible side effects of adjuvant treatments [1, 2]. Since the introduction of cisplatin-based chemotherapy, germ cell tumors have become an example of a curable solid malignancy even in advanced metastatic disease. In metastatic non-seminomas prognosis in all three groups according to the IGCCCG classification as well as the good and intermediate prognosis in seminoma improved over time according to two recent large retrospective analyses [3, 4].

In patients with relapses following primary treatment, two scenarios demand attention: (i) patients with recurrent disease during active surveillance for CS I disease or after adjuvant chemotherapy in case of high-risk parameters and (ii) patients with relapse after first-line cisplatin-based combination chemotherapy for metastatic disease. Although chemotherapy in case of recurrent disease from CS I disease is the standard choice in non-seminomas, retroperitoneal lymph node dissection (RPLND) can be effectively applied in a carefully selected subset of patients. Ongoing studies also assess the option of surgery in seminomas with retroperitoneal relapse (CS II). Treatment of relapses after cisplatin-based chemotherapy and potential residual mass resection is more challenging. Patients are treated either with conventional salvage cisplatin-based chemotherapy regimens or by high-dose chemotherapy.

## Methods

This narrative review is based on a comprehensive literature search of the MEDLINE online repository (via Ovid) and Cochrane Central Register of Controlled Trials which was conducted for the first German clinical practice guideline on testicular cancer. Prospective clinical trials and retrospective studies published between 1990 and 2021 reporting on the use of surgical or chemotherapeutic first salvage treatment for relapsed testicular cancer were identified. Studies were eligible for inclusion if they reported on patients with recurrent testicular cancer (seminomatous and/or non-seminomatous disease) either (i) following active surveillance or adjuvant systemic treatment for clinical stage I (CS I) or (ii) after failure of primary cisplatin-based first-line chemotherapy for advanced metastatic disease (CS ≥ IIC). Endpoints of interests were progression-free (PFS) or relapse-free (RFS) and overall survival (OS), recurrence rates (RR), and safety. The search was restricted to article published in English or German language. Further, ESMO and ASCO conference proceedings from 2019 to 2021 were searched to identify studies, which have not been published completely so far. Case reports, case series, and editorial comments were excluded. An additional search for unpublished data and ongoing trial was conducted in clinical trial registries (https://www.clinicaltrials.gov/).

## Results

### Relapse in patients initially treated for clinical stage I disease

Guideline-endorsed treatment of choice for patients with clinical stage I seminoma and non-seminomatous germ cell cancer is active surveillance, which provides excellent cure rates while avoiding side effects of available adjuvant treatment. For non-seminomatous GCT patients, the risk of relapse from occult metastases is about 30% irrespective of risk factors, and with respect to the absence or presence of lymphovascular invasion 15–20% or 50%, respectively [5–9]. In seminomas the risk of relapse for CS I patients is about 15–25% regardless of risk factors [10, 11] and ranges from 13 to 14% for patients without risk factors and 23–24% for a primary tumor size ≥ 4 cm or rete testis invasion [6, 11]. Relapses after CS I most frequently occur in the retroperitoneal lymph nodes and less frequently more widespread metastases occur during the first 2 and 3 years of non-seminoma and seminoma follow-up.

### First treatment for relapses after CS I disease

Relapsing patients are reclassified according to the UICC stage categories and treated according to current guideline recommendations for primary metastatic disease after IGCCCG classification. For CS IIA/B at relapse these include surgery or chemotherapy for non-seminomas and radiotherapy or chemotherapy or a combination of both for seminomas [12–15].

Since reducing the treatment burden while maintaining treatment success with respect to treatment-related long-term sequelae is of utmost importance in young GCT patients, surgical approaches sparing chemotherapy and radiotherapy in CS IIA/B patients are of particular interest.

### Surgery for recurrent non-seminoma patients on active surveillance

In CS I and CS II non-seminomatous GCTs retroperitoneal lymph node dissection can be seen as a staging and curative approach [16]. Consequently, chemotherapy is not automatically the treatment of choice. In late relapses usually defined after a period of more than two years without tumor marker elevation or patients with a high percentage of teratomatous components in the primary have a significant increased risk of harboring pure teratoma in retroperitoneal manifestations [17, 18] with surgery being the standard of care. Further, to avoid side effects from systemic chemotherapy Hamilton et al. [19] described the results of retroperitoneal lymph node dissection, which is generally recommended for tumor marker-negative late recurrences after chemotherapy. In this large retrospective analysis of prospectively collected patients this treatment approach was investigated in patients with recurrent disease on active surveillance. Of 580 patients with CS I non-seminomatous GCTs, 162 men relapsed during active surveillance. In retroperitoneal recurrences less than 3 cm in short axis diameter, tumor markers S0 and S1 with low doubling time (which is not described in detail), full bilateral nerve sparing retroperitoneal lymph node dissection (RPLND) was performed. In total, 45 patients (72.6%) of the patients were cured by RPLND alone. On multivariate analysis, only tumor marker elevation was an independent risk factor for subsequent additional treatment (HR6.05; 95%CI1.59–23.09; *p* < 0.0084). Only 17.8% of the patients with negative markers needed further treatment. Even in patients with elevated markers prior to surgery no additional treatment was necessary in half of the patients. There were no cancer-related deaths in the group, although the authors critically remark that those patients had a more favorable disease at relapse. The authors highlighted a significantly lower number of cumulative chemotherapy cycles with a primary RPLND at relapse, which was almost equal to patients who had received one cycle of adjuvant BEP [19].

### Surgery for recurrent seminoma patients on active surveillance or after adjuvant Carboplatin

Data on retroperitoneal lymph node dissection in patients with seminoma concentrate on low volume disease in newly diagnosed disease. Due to the historic and retrospective character, trials are heterogeneous and difficult to interpret [20–22]. The relapse rates ranged from 0 to 67% depending on clinical stage. Patients who had CS I and IIA did not suffer in-field recurrences following surgical primary treatment, out-field relapses occurred in 7% [22]. Meanwhile, there are two prospective trials ongoing, SEMS-Trial, and PRIMETEST (NCT01537548; NCT02797626) in patients with CS IIA-C seminoma. In the PRIMETEST study, not only primary disease cases but also relapses under active surveillance or following adjuvant chemotherapy were included. The rationale for both trials is to reduce the long-term morbidity of radiotherapy, the guideline-endorsed standard in CS IIA, or chemotherapy, the standard of care in clinical stage IIB, through salvage surgery. As no intensified chemotherapy had been applied earlier, there are no desmoplastic reactions; thus, the surgical risks and challenges are comparable to primary RPLND in non-seminomatous GCTs. In both trials, progression-free survival (at 2 or 3 years) is the primary endpoint. As secondary endpoints, quality of life and surgical complication rates are being assessed to obtain data on RPLND safety in low volume primary and recurrent seminoma with retroperitoneal spread only. An interim analysis of the German PRIMETEST trial was presented at the ASCO GU Meeting 2019 with promising results [23]. At this time, 14 patients had been included and after a median follow-up of 12.5 [3–25] months 4 relapses occurred. 12 of the 14 patients had either retroperitoneal progression on active surveillance (*n* = 10) or after adjuvant chemotherapy with carboplatin. Contrarily to historic data, predominantly patients with clinical stage IIA disease relapsed after RPLND. The SEMS trial highlighted a 2-year progression-free survival of 82% in 55 patients [24]. Compared to PRIMETEST only primary surgery was allowed, excluding relapses after adjuvant chemotherapy. The final results of the aforementioned two prospective clinical trials are expected and will probably strengthen the impact of RPLND in CS II seminomas at primary diagnosis and relapse from CS I disease.

RPLND is a challenging surgery, in the primary setting as well as after chemotherapy in metastasized disease and even more in the salvage and desperation surgery situation. As clinical outcomes and negative side effects are significantly improved if performed by experienced surgeons, such kind of treatment should be performed in expert centers [19, 20, 25, 26].

### Chemotherapy for advanced metastatic disease following CS I

Patients relapsing with advanced metastatic disease (≥ IIC) are classified according to the IGCCCG risk classification and treated according to primary metastatic GCTs by sequential cisplatin-based combination chemotherapy [12–15, 27].

Interestingly, in a small retrospective study by Fischer et al., patients relapsing after one to two cycles of adjuvant chemotherapy with bleomycin, etoposide, and cisplatin (BEP) seemed to more frequently suffer late relapses than patients on active surveillance and to have inferior outcomes after systemic salvage treatment compared to historical cohorts of patients undergoing first-line combination chemotherapy for de novo metastatic disease [28]. Despite the fact that 84% of the analyzed patients had a good prognosis according to the IGCCCG classification at first relapse after adjuvant BEP, a second relapse rate of 29% is high. This was discussed to be likely associated with a selection for patients with chemoresistant disease and a delayed onset relapse, which are both known risk factors for unfavorable outcomes.

Thus, adjuvant pre-treatment for CS I non-seminomatous disease may negatively impact the prognosis and success of systemic salvage treatment, but this so far does not imply different, i.e. more intensive, salvage treatment approaches. It should only be respected for patient consultation and planning of post-chemo follow-up.

### Relapse after first-line cisplatin-based chemotherapy for primary metastatic disease

The choice of first salvage treatment after 3–4 cycles of cisplatin-based combination chemotherapy, usually bleomycin, etoposide and cisplatin (BEP) or etoposide, ifosfamide, and cisplatin (VIP), is still a matter of controversial discussion. Salvage strategies comprise either conventional dose or high-dose chemotherapy followed by surgical resection of residual post-chemotherapy masses [12, 14, 15].

### Treatment selection based on clinical characteristics

In a large retrospective analysis, the International Prognostic Factor Study Group (IPFSG) established a risk score identifying five distinct risk groups based on seven clinical characteristics (Table 1). There was a significant difference in 2-year PFS rate and 3-year OS rate between the five risk groups. Patients with a very low risk profile had a favorable 2-year PFS rate of 75.1% and 3-year OS-rate of 77%, while patients with a very high-risk profile had a 2-year PFS-rate of 5.6% and 3-year OS-rate of 6.1% only [29]. The IPFSG score does not yet influence salvage treatment decision making but may be helpful for patient counselling with respect to prognosis and should be determined in every patient. In addition to the IPFSG score, the kinetics of serum tumor marker level decline within the first 6 weeks of salvage chemotherapy does predict outcomes [30] as it is the case for patients with primary metastatic disease undergoing first-line cisplatin-based combination chemotherapy (Table 2).

**Table 1:** IPFSG Prognostic Score for patients with relapsing non-seminomas and seminomas

Score points					
Parameter	0	1	2	3	Score
Primary site	Gonadal	Extragonadal	–	Mediastinal	
Response to first-line treatment	CR/PRm-	PRm + /SD	PD	–	
PFI (months)	< 3	≤ 3	–	–	
AFP at relapse	Normal	≤ 1.000	> 1.000	–	
HCG at relapse[Unit]	≤ 1.000	> 1.000	–	–	
LBB	No	yes	–	–	

Final scoring based on histology and risk group scores

*AFP* alpha-1-feto protein, *CR* complete remission, *HCG* ß-subunit of human chorionic gonadotropin, *IPFSG* International Prognostic Factor Study Group, *LBB* presence of liver, brain or bone metastasis, *PRm* + marker-positive partial remission, *PRm-* marker-negative partial remission, *PFI* progression-free interval, *SD* stable disease

**Table 2:** Prognostic score in relapsing testis cancer patients

Prognostic risk group	Score	2-year PFS (%)	3-year OS (%)
Very low	− 1	75.1	77.0
Low	0	51.0	65.6
Intermediate	1	40.1	58.3
High	2	25.9	27.1
Very high	3	5.6	6.1

*OS* overall survival, *PFS* progression-free survival

### Conventional salvage chemotherapy

Long-lasting remissions following first salvage conventional dose cisplatin-based chemotherapy can be achieved in about 15–60% of patients [31]. Conventional dose cisplatin-based combination chemotherapy with cisplatin, ifosfamide, and either paclitaxel (TIP) [32, 33], etoposide (VIP) [34] or vinblastine (VeIP) [35]) is currently the guideline-endorsed first salvage treatment option of choice [12, 14]. The complete secondarily resection of all visible residual masses, including those with a diameter < 1 cm, is essential for treatment success, particularly in non-seminoma patients, due to an increased frequency of residual viable GCT components resected post-salvage chemotherapy masses. TIP is the most frequently used combination, but randomized comparisons between either of these combinations are lacking.

In a prospective study including 46 GCT patients at first relapse, 32 (70%) achieved a complete remission following TIP treatment (29 with TIP alone, 3 with subsequent complete residual mass resection), three of whom relapsed again. Interestingly, TIP was also very active in 14 late relapsing patients, of whom 50% achieved a CR following TIP ± secondary residual mass resection and no further relapses in the patients who achieved a CR. The 2-year PFS rate and 2-year OS rate were 65% (95 %CI 51–79%) and 78% (95% CI 66–90%) [33]. Dosage and efficacy of currently used conventional dose, and cisplatin-based salvage chemotherapy regimens are listed in Table 3.

**Table 3:** First salvage chemotherapy regimens after failure of first-line cisplatin-based combination chemotherapy

Regimen	Dose	Days	CR rate	*N* patients	References
TIP					
Paclitaxel Ifosfamide Cisplatin	175–200 mg/m^2^ 1200 mg/m^2^ 20 mg/m^2^	1 1–5 1–5	70%	46	Kondagunta [33]
VIP					
Etoposide Ifosfamide Cisplatin	75 mg/m^2^ 1200 mg/m^2^ 20 mg/m^2^	1–5 1–5 1–5	25%	42	Motzer [34]
VeIP					
Vinblastine Ifosfamide Cisplatin	0.11 mg/m^2^ 1200 mg/m^2^ 20 mg/m^2^	1 + 2 1–5 1–5	50%	135	Loehrer [35]
HD-CE Indiana					
Carboplatin Etoposide PBSCT	700 mg/m^2^ 750 mg/m^2^ 1 × 10^6^ CD34 + /kg	1–3 1–3 6	63%	184	Einhorn [38]
HD-CE MSKCC					
Carboplatin %Etoposide PBSCT	AUC8 400 mg/m^2^ 2 × 10^6^ CD34 + /kg	1–3 1–3 5	55%	48	Kondagunta [39]

*CR* complete remission, *MSKCC* Memorial Sloan Kettering Cancer Center, *NR* not reported, *PBSCT* autologous peripheral blood stem cell transplantation, *PFS* progression-free survival

### High-dose chemotherapy

The use of salvage high-dose chemotherapy at first relapse of patients with primary metastatic disease is still a matter of debate. There are several retrospective studies and one prospective randomized clinical trial, which evaluated the activity of first salvage high-dose chemotherapy, to date.

When salvage high-dose chemotherapy is chosen, the combination of carboplatin and etoposide is the combination of choice. Before dose-intensified treatment, mobilization chemotherapy, usually consisting of one or two cycles of paclitaxel and ifosfamide (TI), is applied prior stem cell apheresis. TIP seems to be inferior to TI with respect to stem mobilization capability [36].

In the only published randomized clinical trial so far, the IT-94 trial, a high-dose chemotherapy approach consisting of three cycles VeIP or VIP followed by a single consolidating cycle of HD-CE, was compared to four cycles of conventional salvage chemotherapy with VeIP or VIP [37]. Of 263 patients randomized, 128 received standard therapy and 135 patients high-dose chemotherapy. The objective response rate was insignificantly higher in the high-dose arm (67% versus 75%; *p* = 0.23), as was the 3-year PFS rate (55% versus 75%; *p* = 0.04), but without translation into an OS benefit (53% in both arms after 5 years). As prognostic factors for OS, primary tumor localization (*p* < 0.001), initial complete response (*p* = 0.009), absence of pulmonary metastases (*p* < 0.001), number of recurrences (*p* < 0.001), and LDH levels (*p* < 0.001) but not the kind of treatment (*p* = 0.92). Single high-dose chemotherapy has therefore not been adopted as standard salvage approach for relapsing advanced/metastatic GCTs.

Instead, two different regimens of sequential high-dose treatment are commonly used: either two or three consecutive cycles of three days of HD-CE each followed by autologous stem cell transplantation with repetition after blood count recovery every 3–4 or 2–3 weeks, respectively [38, 39].

The Indiana University protocol consists of two cycles of carboplatin (700 mg/m^2^, days 1–3) and etoposide (700 mg/m^2^, days 1–3) followed by autologous stem cell transplantation after prior stem cell mobilization with granulocyte colony formation factor application and an optional preceding cycle of VeIP in case of disease progression or bulky disease [38]. In a recent retrospective study including 364 patients treated with salvage high-dose chemotherapy at first or second relapse at Indiana University, the 2-year PFS and OS rates were 60% (95% CI 55–65%) and 66% (95% CI 60–70%), respectively. High-dose treatment as first salvage resulted in a 2-year PFS rate of 63% (95% CI 57–68%) and as second salvage in a 2-year PFS of 49% (95% CI 36–61%). The treatment-related mortality was 2.5% [40]. The Memorial Sloan Kettering Cancer Centre (MSKCC) protocol consists of two cycles of conventionally dosed mobilization chemotherapy with TI followed by three sequential cycles of carboplatin (AUC8, days 1–3) and etoposide (400 mg/m^2^, days 1–3) plus autologous stem cell transplantation on day 5 (TI-CE)[41]. In a prospective phase II study including 48 consecutive patients, complete remissions were achieved in 49% of patients with salvage TI-CE alone and a further 6% after secondary residual mass resection. 51% of patients were relapse-free after a median follow-up of 40 months [39] (also see Table 3). A following prospective analysis of 107 patients undergoing TI-CE as first or second salvage approach confirmed a complete remission rate of 50% with a 5-year disease-free survival and OS of 47% and 52%. All progression events after TI-CE occurred within 2 years from the start of salvage treatment [41].

Although a randomized comparison between sequential HD-CE and conventional chemotherapy is currently lacking, comparative retrospective studies suggest a beneficial impact of salvage HD-CE on patient survival. In the largest comparative assessment so far based on the IPFSG database, a post hoc matched subgroup analysis of 1594 patients found that patients of all five IPFSG subgroups benefitted from intensified first salvage treatment with sequential high-dose chemotherapy in terms of a 10–15% OS improvement. Across all risk groups, patients undergoing salvage high-dose chemotherapy had improved 2-year PFS and 5-year OS rates (PFS: HR 0.44; 95% CI 0.39–0.51; OS: HR 0.65; 95% CI 0.56–0.75) [31]. In another retrospective analysis of 95 patients undergoing high-dose chemotherapy versus 48 patients receiving conventional salvage chemotherapy, the detected inferior PFS following conventional dose chemotherapy (8 versus 42 months, *p* < 0.001) was associated with a higher rate of vital tumor components in residual mass resection specimens (75% versus 44%). However, OS rates did not differ between both treatment groups (*p* = 0.931) [42]. Notably, patients receiving high-dose chemotherapy had worse prognostic factors, i.e., more frequent non-pulmonary visceral metastases (*p* = 0.032), HCG elevations (*p* = 0.022), and worse responses to first-line chemotherapy, which may account for the similar OS rates. In a more homogeneous, but even smaller retrospective matched-pair analysis published by Beyer et al. [43], a total of 55 patients relapsing after primary chemotherapy, who underwent conventional salvage chemotherapy were matched to 55 patients undergoing high-dose chemotherapy. A significant benefit with respect to PFS (HR 0.72 (95% CI 0.59–0.87; *p* < 0,001)) and OS (HR 0.75 (95% CI 0.61–0.92; *p* = 0,004)) was detected in favor of the high-dose salvage treatment approach.

Several studies did assess the use of triplet high-dose chemotherapy combinations by adding a third drug, i.e., ifosfamide [44, 45], cyclophosphamide [46, 47], or thiotepa [36] resulting in added toxicity and increased treatment-related mortality. In a randomized trial, sequential high-dose chemotherapy with three cycles of HD-CE was compared to three conventional cycles of VIP and one cycle of high-dose CE plus cyclophosphamide (HD-CEC). Due to an unacceptable toxicity with a significantly increased treatment-related death rate in the investigational arm, the trial closed prematurely. Consequently, triplet combinations are not considered for high-dose chemotherapy in refractory GCT patients.

Based on the limited level of evidence and the lack of prospective randomized trial results showing a clear benefit from dose-intensified salvage chemotherapy, the use of high-dose chemotherapy has not uniformly been adopted as first salvage standard of care for all patients, so far. However, conventional and high-dose chemotherapy are both acceptable options for first salvage treatment [14]. Still, the superiority of first salvage high-dose chemotherapy in terms of PFS and OS across almost all defined IPFSG risk groups in a large retrospective matched pair comparison is a strong surrogate of (i) the continued exceptional chemo sensitivity even of relapsed GCTs and (ii) the potential of high-dose chemotherapy to substantially improve patient outcomes. The German S3 Clinical Practice Guideline was the first to recommend first salvage high-dose chemotherapy as the preferred approach, particularly for patients with bone or brain metastases [15].

Whenever possible, relapsing patients should be included in clinical trials, i.e., the ongoing prospective phase III TIGER trial (NCT02375204). In this trial, patients are being randomly assigned to either first salvage treatment with four cycles of conventional dose TIP or two cycles of TI followed for stem cell mobilization and subsequent high-dose chemotherapy with three consecutive cycles of carboplatin and etoposide plus autologous stem cell transplantation (TI-CE). This study will soon clarify whether or not dose-intensified treatment is superior to conventional dose chemotherapy in the first salvage setting. Generally, it is strongly recommended that GCT patients at first relapse are referred to GCT expert centers to provide expert advice on the optimal individual salvage approach and to minimize treatment-related mortality for those undergoing high-dose chemotherapy.

## Conclusion

Patients relapsing after primary treatment for CS I disease are re classified according to UICC and in case of advanced metastatic disease (C SIIC-III) subsequently according to the IGCCCG risk classification. Treatment recommendations comprise the standard first-line treatment for such stages at primary diagnosis without any adaption respecting possible prior adjuvant treatment after orchidectomy.

First salvage treatment options for relapses after cisplatin-based combination chemotherapy for primary advanced metastatic disease include both conventional dose cisplatin-based chemotherapy and salvage high-dose chemotherapy plus autologous stem cell transplantation.

Although the only prospective randomized trial so far failed to demonstrate a survival benefit in patients receiving a single consolidation cycle of HD-CE, sequential HD-CE holds promise to substantially improve patient OS. Whenever GCT experts recommend the use of high-dose chemotherapy, two or three cycles of HD-CE represent the treatment of choice. Patients with testis cancer and recurrent disease are still treated with curative intention if treated properly. To achieve best treatment outcome patients need to be transferred to experienced centers to offer systemic treatment, timely surgical interventions, and treatment in clinical trials.

## References

[1] Tandstad T, Stahl O, Hakansson U, Dahl O, Haugnes HS, Klepp OH (2014). One course of adjuvant BEP in clinical stage I nonseminoma mature and expanded results from the SWENOTECA group. Ann Oncol.

[2] Pierorazio PM, Albers P, Black PC, Tandstad T, Heidenreich A, Nicolai N (2018). Non-risk-adapted surveillance for stage I testicular cancer: critical review and summary. Eur Urol.

[3] Beyer J, Collette L, Sauvé N, Daugaard G, Feldman DR, Tandstad T (2021). Survival and new prognosticators in metastatic seminoma: results from the IGCCCG-update consortium. JCO.

[4] Gillessen S, Sauvé N, Collette L, Daugaard G, de Wit R, Albany C (2021). Predicting outcomes in men with metastatic nonseminomatous germ cell tumors (NSGCT): results from the IGCCCG update consortium. JCO.

[5] Tandstad T, Smaaland R, Solberg A, Bremnes RM, Langberg CW, Laurell A (2011). Management of seminomatous testicular cancer: a binational prospective population-based study from the Swedish Norwegian testicular cancer study group. JCO.

[6] Kollmannsberger C, Tandstad T, Bedard PL, Cohn-Cedermark G, Chung PW, Jewett MA (2015). Patterns of relapse in patients with clinical stage I testicular cancer managed with active surveillance. JCO.

[7] Aparicio J, Maroto P, del Muro XG, Guma J, Sanchez-Munoz A, Margeli M (2011). Risk-adapted treatment in clinical stage I testicular seminoma: the third Spanish Germ Cell Cancer Group study. JCO.

[8] Mortensen MS, Bandak M, Kier MG, Lauritsen J, Agerbaek M, Holm NV (2017). Surveillance versus adjuvant radiotherapy for patients with high-risk stage I seminoma. Cancer.

[9] van de Wetering RAW, Sleijfer S, Feldman DR, Funt SA, Bosl GJ, de Wit R (2018). Controversies in the management of clinical stage I seminoma: carboplatin a decade in-time to start backing out. JCO.

[10] Mortensen MS, Lauritsen J, Gundgaard MG, Agerbaek M, Holm NV, Christensen IJ (2014). A nationwide cohort study of stage I seminoma patients followed on a surveillance program. Eur Urol.

[11] Warde P, Specht L, Horwich A, Oliver T, Panzarella T, Gospodarowicz M (2002). Prognostic factors for relapse in stage I seminoma managed by surveillance: a pooled analysis. JCO.

[12] Albers P, Albrecht W, Algaba F, Bokemeyer C, Cohn-Cedermark G, Fizazi K (2015). Guidelines on testicular cancer: 2015 update. Eur Urol.

[13] Gilligan T, Lin DW, Aggarwal R, Chism D, Cost N, Derweesh IH (2019). Testicular cancer, version 2.2020, NCCN Clinical Practice Guidelines in Oncology. J Natl Compr Canc Netw.

[14] Honecker F, Aparicio J, Berney D, Beyer J, Bokemeyer C, Cathomas R, et al (2018) ESMO Consensus Conference on testicular germ cell cancer: diagnosis, treatment and follow-up. Ann Oncol 29(8):1658–1686. 10.1093/annonc/mdy217 30113631

[15] Kliesch S, Schmidt S, Wilborn D, Aigner C, Albrecht W, Bedke J (2021). Management of germ cell tumours of the testes in adult patients: German Clinical Practice Guideline, PART II—recommendations for the treatment of advanced, recurrent, and refractory disease and extragonadal and sex cord/stromal tumours and for the management of follow-up, toxicity, quality of life, palliative care, and supportive therapy. Urol Int.

[16] Stephenson AJ, Bosl GJ, Motzer RJ, Kattan MW, Stasi J, Bajorin DF (2005). Retroperitoneal lymph node dissection for nonseminomatous germ cell testicular cancer: impact of patient selection factors on outcome. JCO.

[17] Heidenreich A, Moul JW, McLeod DG, Mostofi FK, Engelmann UH (1997). The role of retroperitoneal lymphadenectomy in mature teratoma of the testis. J Urol.

[18] Leibovitch I, Foster RS, Ulbright TM, Donohue JP (1995). Adult primary pure teratoma of the testis. Indiana Exp Cancer.

[19] Hamilton RJ, Nayan M, Anson-Cartwright L, Atenafu EG, Bedard PL, Hansen A (2019). Treatment of relapse of clinical stage I nonseminomatous germ cell tumors on surveillance. JCO.

[20] Hu B, Shah S, Shojaei S, Daneshmand S (2015). Retroperitoneal lymph node dissection as first-line treatment of node-positive seminoma. Clin Genitourin Cancer.

[21] Mezvrishvili Z, Managadze L (2006). Retroperitoneal lymph node dissection for high-risk stage I and stage IIA seminoma. Int Urol Nephrol.

[22] Warszawski N, Schmucking M (1997). Relapses in early-stage testicular seminoma: radiation therapy versus retroperitoneal lymphadenectomy. Scand J Urol Nephrol.

[23] Albers P, Hiesters A, Grosse-Siemer R (2019). The PRIMETEST trial: Interim analysis of a phase II trial for primary retroperitoneal lymph node dissection (RPLND) in stage II A/B seminoma patients without adjuvant treatment. JCO.

[24] Daneshmand S (2021). SEMS trial: result of a prospective, multi-institutional phase II clinical trial of surgery in early metastatic seminoma treatment. JCO.

[25] Albers P, Siener R, Krege S, Schmelz HU, Dieckmann KP, Heidenreich A (2008). Randomized phase III trial comparing retroperitoneal lymph node dissection with one course of bleomycin and etoposide plus cisplatin chemotherapy in the adjuvant treatment of clinical stage I Nonseminomatous testicular germ cell tumors: AUO trial AH 01/94 by the German Testicular Cancer Study Group. Journal of clinical oncology : official journal of the American Society of Clinical Oncology.

[26] Heidenreich A, Albers P, Hartmann M, Kliesch S, Kohrmann KU, Krege S (2003). Complications of primary nerve sparing retroperitoneal lymph node dissection for clinical stage I nonseminomatous germ cell tumors of the testis: experience of the German Testicular Cancer Study Group. J Urol.

[27] International Germ cell cancer collaborative Group (1997). International Germ Cell Consensus Classification: a prognostic factor-based staging system for metastatic germ cell cancers. J Clin Oncol Off J Am Soc Clin Oncol.

[28] Fischer S, Tandstad T, Cohn-Cedermark G, Thibault C, Vincenzi B, Klingbiel D (2020). Outcome of men with relapses after adjuvant bleomycin, etoposide, and cisplatin for clinical stage I nonseminoma. JCO.

[29] Lorch A, Beyer J, Bascoul-Mollevi C, Kramar A, Einhorn LH, Necchi A (2010). Prognostic factors in patients with metastatic germ cell tumors who experienced treatment failure with cisplatin-based first-line chemotherapy. JCO.

[30] Massard C, Kramar A, Beyer J, Hartmann JT, Lorch A, Pico JL (2013). Tumor marker kinetics predict outcome in patients with relapsed disseminated non-seminomatous germ-cell tumors. Ann Oncol.

[31] Lorch A, Bascoul-Mollevi C, Kramar A, Einhorn L, Necchi A, Massard C (2011). Conventional-dose versus high-dose chemotherapy as first salvage treatment in male patients with metastatic germ cell tumors: evidence from a large international database. JCO.

[32] Motzer RJ, Sheinfeld J, Mazumdar M, Bains M, Mariani T, Bacik J (2000). Paclitaxel, ifosfamide, and cisplatin second-line therapy for patients with relapsed testicular germ cell cancer. JCO.

[33] Kondagunta GV, Bacik J, Donadio A, Bajorin D, Marion S, Sheinfeld J (2005). Combination of paclitaxel, ifosfamide, and cisplatin is an effective second-line therapy for patients with relapsed testicular germ cell tumors. JCO.

[34] Motzer RJ, Cooper K, Geller NL, Bajorin DF, Dmitrovsky E, Herr H (1990). The role of ifosfamide plus cisplatin-based chemotherapy as salvage therapy for patients with refractory germ cell tumors. Cancer.

[35] Loehrer PJ, Gonin R, Nichols CR, Weathers T, Einhorn LH (1998). Vinblastine plus ifosfamide plus cisplatin as initial salvage therapy in recurrent germ cell tumor. JCO.

[36] Rick O, Bokemeyer C, Beyer J, Hartmann JT, Schwella N, Kingreen D (2001). Salvage treatment with paclitaxel, ifosfamide, and cisplatin plus high-dose carboplatin, etoposide, and thiotepa followed by autologous stem-cell rescue in patients with relapsed or refractory germ cell cancer. JCO.

[37] Pico JL, Rosti G, Kramar A, Wandt H, Koza V, Salvioni R (2005). A randomised trial of high-dose chemotherapy in the salvage treatment of patients failing first-line platinum chemotherapy for advanced germ cell tumours. Ann Oncol.

[38] Einhorn LH, Williams SD, Chamness A, Brames MJ, Perkins SM, Abonour R (2007). High-dose chemotherapy and stem-cell rescue for metastatic germ-cell tumors. N Engl J Med.

[39] Kondagunta GV, Bacik J, Sheinfeld J, Bajorin D, Bains M, Reich L (2007). Paclitaxel plus Ifosfamide followed by high-dose carboplatin plus etoposide in previously treated germ cell tumors. JCO.

[40] Adra N, Abonour R, Althouse SK, Albany C, Hanna NH, Einhorn LH (2017). High-dose chemotherapy and autologous peripheral-blood stem-cell transplantation for relapsed metastatic germ cell tumors: the Indiana university experience. JCO.

[41] Feldman DR, Sheinfeld J, Bajorin DF, Fischer P, Turkula S, Ishill N (2010). TI-CE high-dose chemotherapy for patients with previously treated germ cell tumors: results and prognostic factor analysis. JCO.

[42] Berger LA, Bokemeyer C, Lorch A, Hentrich M, Kopp HG, Gauler TC (2014). First salvage treatment in patients with advanced germ cell cancer after cisplatin-based chemotherapy: analysis of a registry of the German Testicular Cancer Study Group (GTCSG). J Cancer Res Clin Oncol.

[43] Beyer J, Stenning S, Gerl A, Fossa S, Siegert W (2002). High-dose versus conventional-dose chemotherapy as first-salvage treatment in patients with non-seminomatous germ-cell tumors: a matched-pair analysis. Ann Oncol.

[44] Broun ER, Nichols CR, Tricot G, Loehrer PJ, Williams SD, Einhorn LH (1991). High dose carboplatin/VP-16 plus ifosfamide with autologous bone marrow support in the treatment of refractory germ cell tumors. Bone Marrow Transplant.

[45] Siegert W, Beyer J, Strohscheer I, Baurmann H, Oettle H, Zingsem J (1994). High-dose treatment with carboplatin, etoposide, and ifosfamide followed by autologous stem-cell transplantation in relapsed or refractory germ cell cancer: a phase I/II study. The German Testicular Cancer Cooperative Study Group. JCO.

[46] Lorch A, Kleinhans A, Kramar A, Kollmannsberger CK, Hartmann JT, Bokemeyer C (2012). Sequential versus single high-dose chemotherapy in patients with relapsed or refractory germ cell tumors: long-term results of a prospective randomized trial. JCO.

[47] Motzer RJ, Mazumdar M, Bosl GJ, Bajorin DF, Amsterdam A, Vlamis V (1996). High-dose carboplatin, etoposide, and cyclophosphamide for patients with refractory germ cell tumors: treatment results and prognostic factors for survival and toxicity. JCO.

